# CT-free attenuation and Monte-Carlo based scatter correction-guided quantitative ^90^Y-SPECT imaging for improved dose calculation using deep learning

**DOI:** 10.1007/s00259-025-07191-5

**Published:** 2025-03-13

**Authors:** Zahra Mansouri, Yazdan Salimi, Nicola Bianchetto Wolf, Ismini Mainta, Habib Zaidi

**Affiliations:** 1 Division of Nuclear Medicine and Molecular Imaging, Geneva University Hospital, Geneva, CH-1211 Switzerland; 2 Department of Nuclear Medicine and Molecular Imaging, University of Groningen, University Medical Center Groningen, Groningen, Netherlands; 3 Department of Nuclear Medicine, University of Southern Denmark, Odense, Denmark; 4 University Research and Innovation Center, Óbuda University, Budapest, Hungary

**Keywords:** SPECT/ CT, Attenuation correction, Scatter correction, Deep learning, ^90^Y dosimetry

## Abstract

**Background:**

This work aimed to develop deep learning (DL) models for CT-free attenuation and Monte Carlo-based scatter correction (AC, SC) in quantitative ^90^Y SPECT imaging for improved dose calculation.

**Methods:**

Data of 190 patients who underwent ^90^Y selective internal radiation therapy (SIRT) with glass microspheres was studied. Voxel-level dosimetry was performed on uncorrected and corrected SPECT images using the local energy deposition method. Three deep learning models were trained individually for AC, SC, and joint ASC using a modified 3D shifted-window UNet Transformer (Swin UNETR) architecture. Corrected and unorrected dose maps served as reference and as inputs, respectively. The data was split into train set (~ 80%) and unseen test set (~ 20%). Training was conducted in a five-fold cross-validation scheme. The trained models were tested on the unseen test set. The model’s performance was thoroughly evaluated by comparing organ- and voxel-level dosimetry results between the reference and DL-generated dose maps on the unseen test dataset. The voxel and organ-level evaluations also included Gamma analysis with three different distances to agreement (DTA (mm)) and dose difference (DD (%)) criteria to explore suitable criteria in SIRT dosimetry using SPECT.

**Results:**

The average ± SD of the voxel-level quantitative metrics for AC task, are mean error (ME (Gy)): -0.026 ± 0.06, structural similarity index (SSIM (%)): 99.5 ± 0.25, and peak signal to noise ratio (PSNR (dB)): 47.28 ± 3.31. These values for SC task are − 0.014 ± 0.05, 99.88 ± 0.099, 55.9 ± 4, respectively. For ASC task, these values are as follows: -0.04 ± 0.06, 99.57 ± 0.33, 47.97 ± 3.6, respectively. The results of voxel level gamma evaluations with three different criteria, namely “DTA: 4.79, DD: 1%”, “DTA:10 mm, DD: 5%”, and “DTA: 15 mm, DD:10%” were around 98%. The mean absolute error (MAE (Gy)) for tumor and whole normal liver across tasks are as follows: 7.22 ± 5.9 and 1.09 ± 0.86 for AC, 8 ± 9.3 and 0.9 ± 0.8 for SC, and 11.8 ± 12.02 and 1.3 ± 0.98 for ASC, respectively.

**Conclusion:**

We developed multiple models for three different clinically scenarios, namely AC, SC, and ASC using the patient-specific Monte Carlo scatter corrected and CT-based attenuation corrected images. These task-specific models could be beneficial to perform the essential corrections where the CT images are either not available or not reliable due to misalignment, after training with a larger dataset.

**Supplementary Information:**

The online version contains supplementary material available at 10.1007/s00259-025-07191-5.

## Introduction

Selective internal radiation therapy (SIRT) is a treatment option for primary or secondary liver cancer, where microspheres containing ^90^Yttrium (^90^Y) are injected directly into the hepatic artery, lodged in the tumoural microvasculature, to irradiate internally cancer cells with β^−^ particles [1]. ^90^Y is considered as a high-energy β^−^ emitter with a maximum energy of ~ 2.28 MeV, average energy of ~ 0.937 MeV, maximum penetration range in soft tissue of 12 mm and average range of 2.5 mm [2]. The ^90^Y distribution can be monitored post-therapy using bremsstrahlung SPECT (bSPECT) or positron emission tomography (PET) imaging [3].

While state-of-the-art PET offers superior spatial resolution, bSPECT imaging still remains a widely used tool for post-therapy treatment verification due to its broader accessibility and lower cost compared to PET [4, 5]. A key challenge in ^90^Y-bSPECT imaging is fast and accurate correction for physical degrading factors, such as attenuation and scatter, to ensure reliable quantification, an essential component for accurate dosimetry. This process relies on a connected chain, where each step depends on others. Any break in the chain can compromise treatment verification, disrupt dose–response analysis [6], and limit future therapy options, such as SIRT re-treatment or combination with external beam radiotherapy [7–9].

Attenuation correction in SPECT is essential to account for the absorption or scattering of photons as they pass through different tissue types and pathlengths. Photon attenuation within the body leads to reconstruction artefacts and inaccurate quantification [10, 11]. Specifically in SIRT, studies have reported dosimetric relative absolute errors of approximately 15% for tumours and 17% for whole normal liver (WNL) on a voxel-wise basis, and around 10% for both on a region-wise basis, when attenuation correction is not applied in ⁹⁰Y-bSPECT imaging [12]. CT-based attenuation correction is commonly used on hybrid SPECT/CT systems. However, inaccuracies can arise from the propagation of CT artefacts into SPECT images and from misregistration of the two imaging modalities due to respiratory or bulk motion [13].

In clinical setting, scattered photons account for 30–40% of the total photons detected within the photopeak energy window defined during SPECT imaging. This amount of scatter leads to loss of contrast, image distortions and introduces considerable uncertainty in image quantification [14, 15]. Various approaches and strategies were devised for scatter correction (SC) in SPECT imaging [14]. The most commonly used methods in clinical practice are based on multiple-energy window techniques, including the dual-energy window (DEW) [16] and triple-energy window (TEW) techniques [17]. However, these techniques are inefficient for ⁹⁰Y due to the continuous bremsstrahlung energy spectrum and substantial down scatter contamination within the primary energy window.

Monte Carlo (MC) simulations, which fully model the physics of photon transport within the patient and camera, are widely regarded as the gold standard for attenuation and scatter corrections [18–20]. However, MC simulations are computationally demanding and time-consuming and not straightforward for application in clinical settings.

Nowadays, several reconstruction tools are equipped with graphics processing units (GPUs) to implement MC-based reconstruction algorithms, significantly accelerating image reconstruction speed [21, 22]. GPUs offer massive parallel computing capabilities. However, implementing attenuation and scatter modelling on GPUs is challenging due to thread divergence and the complexity of generating pseudorandom numbers, which conflict with the parallel processing nature of GPUs [23]. Moreover, these advanced GPU-based reconstruction tools are expensive and not yet widely available across clinical sites.

With advancements in artificial intelligence, numerous studies have explored the potential of deep learning (DL) algorithms for CT-free attenuation and/or scatter correction in both direct and indirect domain [24, 25]. Promising results have been achieved in both SPECT and PET imaging using a variety of DL architectures [24, 26–31]. Several efforts have specifically focused on developing models for scatter correction in ^90^Y-SIRT, highlighting the significance of this research area [32–34]. However, many of these studies were conducted on limited datasets, raising concerns about the generalizability of the trained models. Moreover, none of the existing studies have developed or evaluated models that address both attenuation and scatter correction simultaneously. There remains a clear need for models with improved performance and broader applicability in this domain.

The aim of this study was to develop dedicated DL models with state-of-the-art architectures for direct attenuation and/or scatter correction, separately or in a combined approach for ⁹⁰Y-bSPECT. Our methodology is designed to address the limitations of Monte Carlo-based corrections, which face an inherent trade-off between accuracy and computational efficiency, as well as the challenges posed by GPU-based reconstructions. To achieve this goal, we trained DL models using a relatively large-scale dataset, with the goal of improving both correction accuracy and speed, to step towards making these advanced techniques more feasible for clinical implementation. In our DL framework, we used “dose maps” from uncorrected and corrected bSPECT images, as input and output of the models, respectively. The approach helped to leverage meaningful quantities of dose values (Gy) instead of raw SPECT counts which reflect a measurement of photon detection.

## Materials and methods

**Fig. 1:**
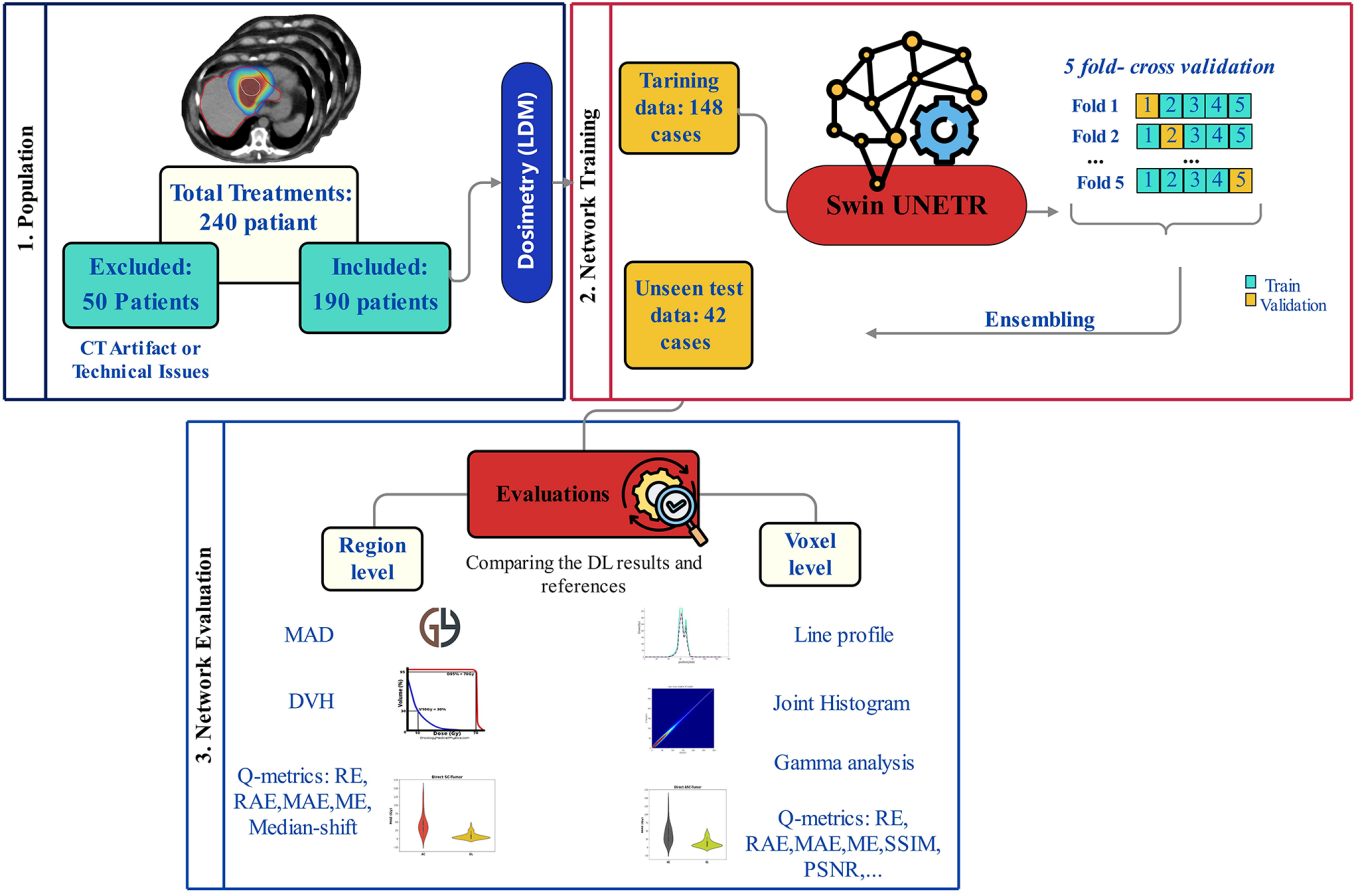
Flowchart of the present study. Overall, 190 patients were included and split into 148 and 42 cases as training and test sets, respectively. corrected and uncorrected images underwent voxel-level dosimetry using the local energy deposition method (LDM). Swin UNETR architecture was adopted and trained in a 5-fold-cross validation approach. The model performance was evaluated at both organ and voxel level. *MAD: mean absorbed dose. DVH: dose-volume histogram, Q-metrics: quantitative metrics

The flowchart of this study is illustrated in Fig. 1.

### Patient population

A relatively large-scale dataset comprising 240 ^90^Y-bSPECT images from patients treated with glass microspheres (TheraSphere™; Boston Scientific, Marlborough, Massachusetts) was included in this retrospective study. Of the 240 cases, 50 cases were excluded owing to the presence of CT artefacts and/or processing technical issues. The remaining 190 patients consisted of 152 males and 38 females. The median age was 68 years (± 12.86 SD, range 26–90), with an average weight of 77.66 kg (± 18.94 SD) and an average height of 1.72 m (± 0.11 SD). The treatments were conducted between January 2011 and January 2023 at Geneva University Hospital (HUG). The mean ± SD of the injected activities of 99mTc-macroaggregated albumin (MAA) was 154.9 ± 8.6 MBq (range 125–190 MBq), according to manufacturer’s guidelines. Planar and SPECT/CT images of ^99m^Tc-MAA were acquired to assess lung shunt fractions and determine therapeutic injection activities. Lung shunt fractions were calculated from planar images, with an average of 7.01% ± 4.2%. Over 12 years of experience with ^90^Y-SIRT at HUG, therapeutic activities were calculated using evolving methodologies, from the single-compartment model to the more recent personalised voxel-wise dosimetry approach. An average of 2.82 ± 1.29 GBq (range 0.63to 6.3 GBq) of ^90^Y-TheraSpheres was administered to the patients.

### ^90^Y SPECT/CT image acquisition

After treatment, ^90^Y bSPECT/CT imaging was performed using two dual-head Symbia-T series cameras (T6 and T16) (Siemens Healthcare Erlangen, Germany). High-energy collimators were employed, and 64 projections were acquired over a 180° angle, with each projection taking between 15 and 30 s, using a 128 × 128 matrix. Co-registered low-dose CT images were obtained at a kVp of either 110 or 130, with an average tube current of 59.39 ± 22.92 mA. CT images were reconstructed in a 512 × 512 matrix, with a pixel size of 0.97 × 0.97 mm and a slice thickness of 3 mm, using a filtered back-projection algorithm.

### Phantom calibration and image reconstruction

SPECT image reconstruction was performed using Hermes HybridRecon™ Oncology version 4.0 (Hermes Medical Solutions Ltd, Stockholm, Sweden). This tool requires defining a conversion factor to convert the counts of SPECT images to activity concentration. This involves using a homogeneous distribution of known activity in a large volume. A uniform cylindrical phantom with a volume of 6200 mL was filled with pure water at approximately 25 °C, ensuring the avoidance of air bubbles. A 605.33 MBq solution of Yttrium-90 Chloride (^90^YCl_3_) was injected directly into the water using a syringe and then mixed thoroughly with magnetic stirring. The MED ISOMED Model 2000/2010 well-type ionisation chamber at HUG, which is verified annually, was utilised for activity measurement. The magnetic stirrer was removed, and the phantom underwent bSPECT/CT imaging using the same protocol used for SIRT patients. The images of the phantom were reconstructed using 4 iterations and 8 subsets, followed by post-reconstruction filtering using a Gaussian filter with a 0.9 cm FWHM. A region of interest covering the central 70% of the phantom was used to estimate the number of counts. The conversion factor was then calculated following Hermes recommendations. Patient data were reconstructed using the same parameters but with three different corrections: (a) uncorrected images (no attenuation and scatter correction, NC), (b) only attenuation correction (AC), and (c) attenuation and scatter correction (ASC). The system does not allow for scatter correction without attenuation correction.

**Fig. 2:**
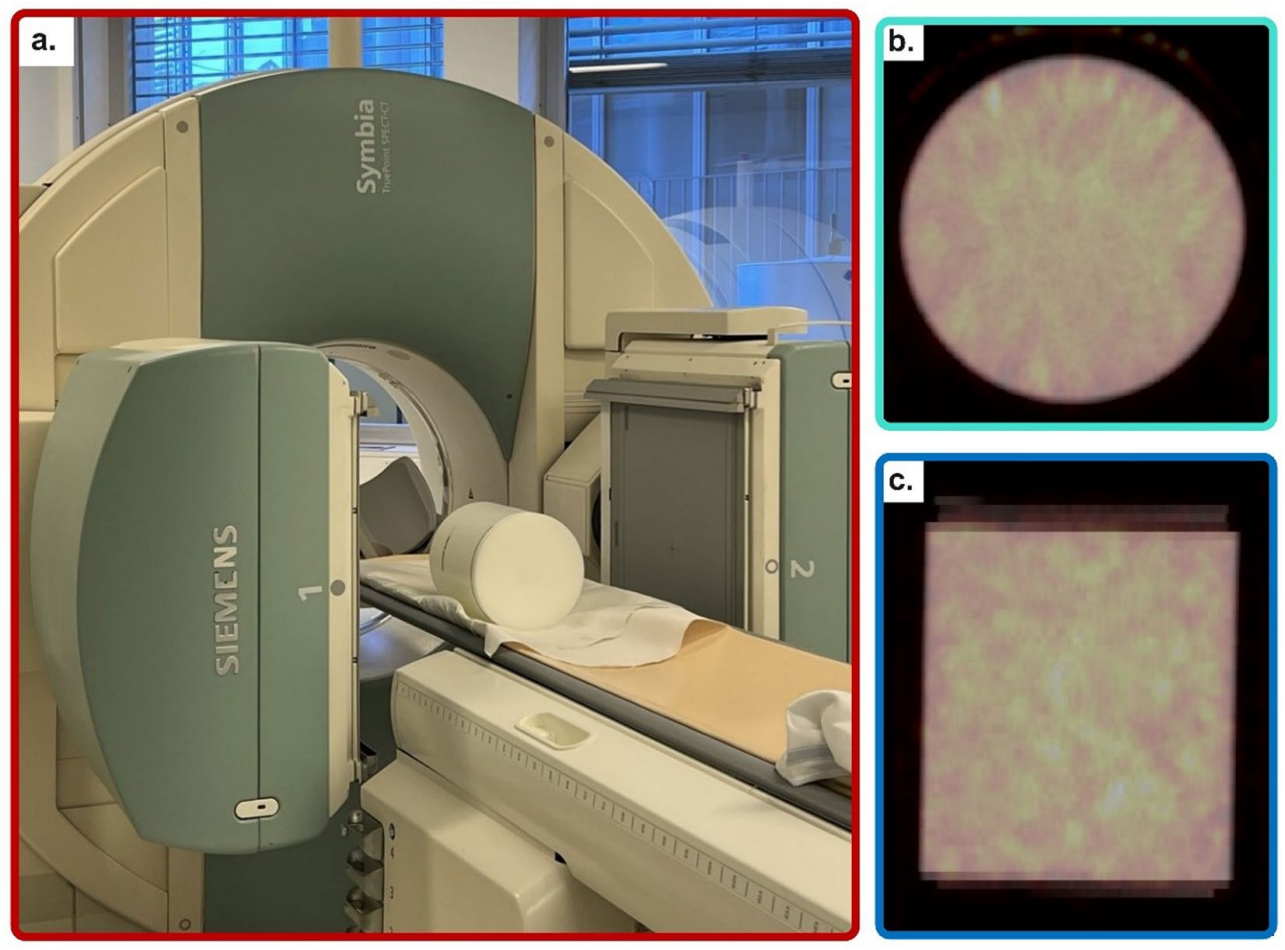
Image acquisition of the calibration phantom. (**a**) Uniform phantom scanning, (**b**) Phantom bSPECT/CT axial view, and (**c**) Coronal view

Notably, HybridRecon SPECT Reconstruction uses a modified Ordered Subsets - Expectation Maximization (OSEM) algorithm and enables attenuation correction (using CT-based attenuation map), scatter correction (using a Monte Carlo-based approach), and collimator and detector blur compensation (using pre-calculated look-up tables) [23, 35–37]. Figure 2 shows the phantom setup in SPECT imaging for calibration purposes.

### Dosimetry calculations

3D voxel-wise dosimetry calculations were performed on NC, AC, and ASC bSPECT images using the local energy deposition method (LDM). LDM assumes that microspheres are permanently trapped within the microvasculature, without biological clearance and with no energy crossfire between voxels. The half-life (T_1/2_) of ^90^Y was set to 64.1 h, with a mean energy of 0.93 MeV, and liver density was set at 1.05 g/cm^3^. To convert image counts into activity distributions, patient-specific calibration was carried out based on the total counts from the whole liver (WL) and the net injected ^90^Y activity. This approach avoids any mismatch between the geometry of the calibration source and the patient’s anatomy, enhancing the accuracy of quantification [38]. Dosimetry calculations were implemented using an in-house Python code, based on details provided in previous studies by our group [39, 40].

As required for our organ-level evaluations, tumoural regions were delineated on contrast-enhanced CT or MR diagnostic images, acquired before treatment. Experienced nuclear medicine physicians manually segmented the tumours. Other structures such as WL, kidneys, lungs, and stomach were delineated using a previously-developed DL-based segmentation model by our group [41]. The WNL was defined by subtracting tumourstructures from the WL delineation.

### Preprocessing and network training

The preprocessing pipeline for loading images into the models included resampling, background removal outside the body contour, and image normalisation. Each image was normalised to a range of zero to one, corresponding to zero as the minimum and 200 Gy as the maximum, without clipping image values. The normalisation values were chosen based on the distribution of the training data.

Three different models were trained for three distinct tasks:

1. Prediction of AC dose maps from NC dose maps (AC task);
2. Prediction of ASC dose maps from AC dose maps (SC task);
3. Prediction of ASC dose maps from NC dose maps (ASC task).

The inputs and outputs for each model are specified in Table 1.

**Table 1:** Input and reference dose images used for training models in distinct tasks: “AC”, “SC”, and “ASC”. *The second row: Since the input images are AC dose maps while the output are ASC dose maps, the task practically performs SC

Task	Model Input	Model Output (reference)
AC	NC dose map	AC dose map
SC*	AC dose map	ASC dose map
ASC	NC dose map	ASC dose map

A modified 3D shifted window (Swin) UNEt TRansformer (Swin UNETR) architecture [42] was employed. This architecture consists of a U-shaped network, with a Swin Transformer as the encoder and a Convolutional Neural Network (CNN) as the decoder. The Swin Transformer generates distinct non-overlapping patches from the input data for self-attention calculations, and the encoded features are transmitted to the CNN via skip connections. We selected the Swin UNETR architecture due to its unique advantages over standard U-Net, ResNet, and GANs, particularly for SPECT attenuation and scatter correction, which require deep contextual understanding and accurate reconstruction. Swin UNETR is more effective because it captures both global and local spatial context, providing superior feature representation for SPECT images. Its self-attention mechanism enhances generalizability compared to convolutional neural networks, such as ResNet and U-Net, which primarily focus on local feature extraction. Additionally, while GAN-based approaches can improve image quality, they can introduce hallucination structures, which is not compliant with the requirements of quantitative imaging.

For each task, patient data was split into 78% (148 cases) for training and 22% (42 cases) for external test sets, respectively. The training set was further divided into 5 folds, with each fold containing an equal number of cases for training in a 5-fold cross-validation framework. In each iteration, data from 80% of training data (80% of 148 ~ 118 cases) were used for internal training, while 20% of training data (20% of 148 ~ 29 cases) were used for testing the model. This approach involves training five different models on the entire training set. The predictions from these trained models were then ensembled by averaging their outputs for each image in the external test group which were unseen to the models during training. For each patient, only one corresponding ^90^Y-SPECT image for each task was used for dosimetry and deep learning implementations.

To improve generalizability and decrease overfitting, we used random patch sampling from each training image and rotated and flipped them. We avoided deformable data augmentation to maintain the quantitative information in the training set. This approach involved cropping or padding different regions of the image to create diverse training samples. In addition, dropout was set to 0.1 and data shuffling was implemented before training to prevent order bias.

Training was performed using 3D patches with a patch size of 64 × 64 × 64 voxels and an original voxel dimension of 4.795 mm. An initial learning rate of 1 × 10⁻³, a weight decay of 1 × 10⁻⁴, and a batch size of 2 were used. The Adam optimizer and L1 loss function (Mean Absolute Error) were adopted, with training continuing for 200 epochs. Network training was implemented in MONAI and run on an NVIDIA GeForce RTX 3080 GPU with 10 GB of dedicated random-access memory (RAM). Test image inference was performed using a sliding window inference method with the same patch size used in training.

It should be mentioned that in our study, two approaches were followed for training the models. The first approach, detailed above, used LDM dose maps as input and output to the models. In the second approach, SPECT images were used, and model performance was evaluated by calculating LDM dose maps from the DL-generated SPECT images. The results and details of the second approach are provided in the supplementary material.

### Network evaluation: organ and voxel-level dosimetry assessments

The performance of the models was evaluated on an unseen test set using both voxel-wise and region-wise dosimetry assessments. For voxel-level assessments, uncorrected (input), and DL-generated results were compared to the reference (output) dose maps. Quantitative metrics for comparison included the structural similarity index (SSIM), root mean squared error (RMSE), peak signal-to-noise ratio (PSNR), mean absolute error (MAE), mean error (ME), relative error (RE), and relative absolute error (RAE). Voxel-wise evaluations were extended to joint histograms, dose map line profiles and 3D gamma analysis between reference and DL-generated dose maps. Gamma evaluations were conducted using a 4.795 mm distance-to-agreement (DTA) and a 1% dose-difference (DD) criterion, along with additional DTA and DD criteria (5% and 10 mm; 10% and 15 mm) to explore suitable criteria for gamma evaluations in SIRT.

For the region-wise absorbed dose analysis, two key regions were of more importance: the tumour and the WNL. Furthermore, we extended our assessments to additional organs, including the kidneys, lungs, and stomach, to evaluate the impact of image correction on organs with varying densities. The same OAR segmentation model was applied for these organs. The segmentations were checked visually and corrected if necessary.

Evaluation metrics such as MAE, ME, RE, RAE, and median shift were calculated for each region. Additionally, the mean absorbed dose (MAD) and dose-volume histograms (DVHs) of each organ were compared across reference, uncorrected, and DL-based results. To compare the mean absorbed doses between approaches, the Wilcoxon Rank-Sum (Mann-Whitney U-test) statistical test was employed.

Also, the AC model was inferenced on the calibration phantom using NC and AC images. We also tested our AC model on an external ^90^Y SPECT-guided dose map with different spacing than our train and test dataset (4.418 × 4.418 × 4.418 with an image size of 128 × 128) acquired on a different camera, the GE NM 860 SPECT/CT.

## Results

### Voxel-level evaluations

**Fig. 3:**
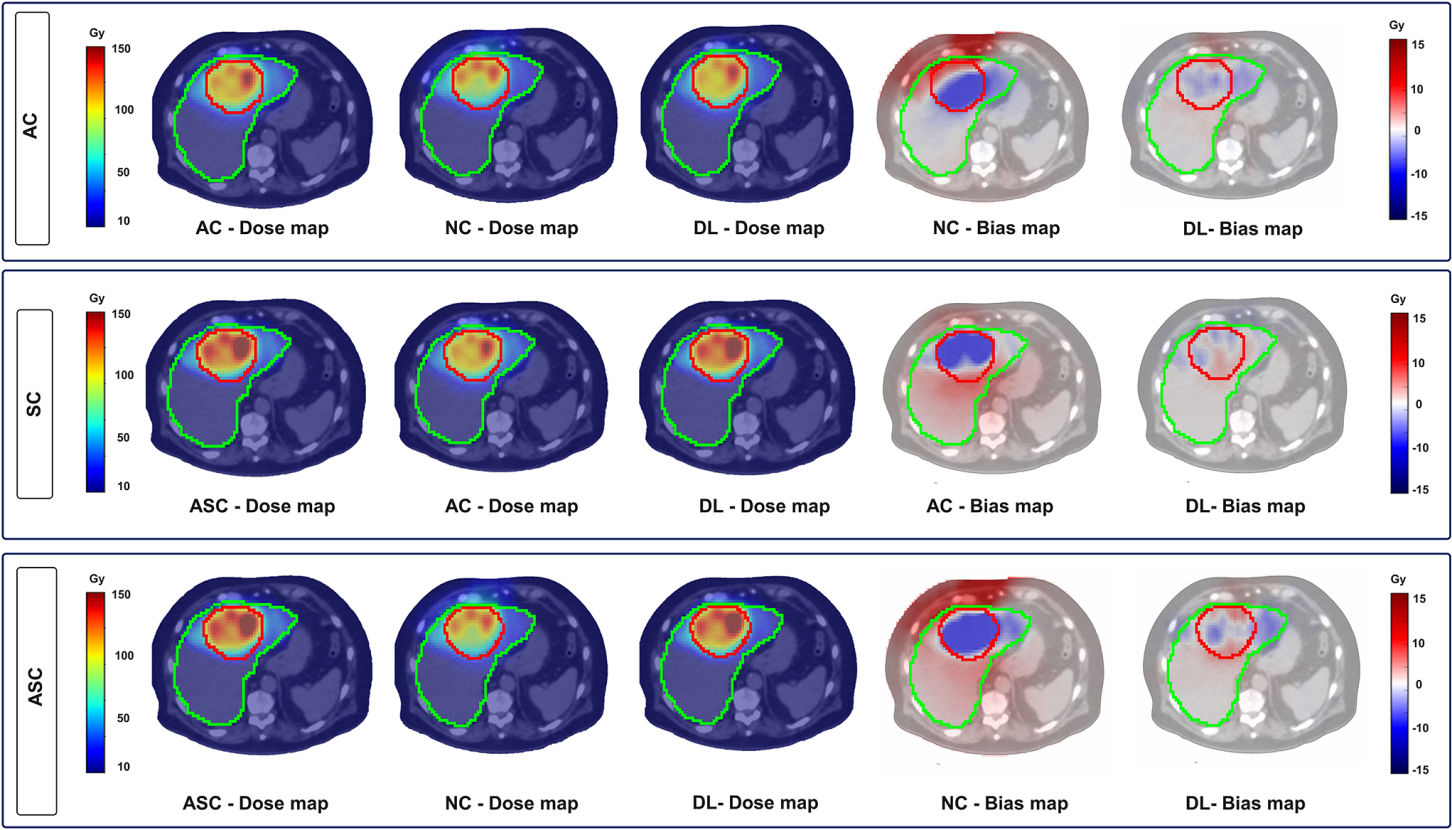
Representative dose maps from the Reference, Input, and DL-results across all three tasks. Bias maps relative to the Reference dose maps are also shown for both the Input and DL-results. The tumour is outlined in red and the whole liver (WNL) in green. The bias maps indicate errors close to zero within both the tumour and WNL for the DL-based images, while the Inputs show the highest error levels. The negative values in the colorbars of the bias maps indicate regions where the reference dose map has higher values than the predicted dose map

Representative dose maps overlaid on CT images from the Reference, Input, and DL results from all three tasks (AC, SC, and ASC) are illustrated in Fig. 3. All dose maps correspond to a single patient (#22) and are presented in the same axial view. In addition, the bias maps with respect to the Reference dose maps, displayed for both the Input (uncorrected) and DL-results, are shown. The results demonstrate that the performance of our DL models is significantly affected by the presence of metal implants within the body, as well as in cases where the tumor is close to calcifications in the hepatic vasculature or adjacent to air-filled regions, such as gastrointestinal gas or lungs. A representative case where model performance was impacted by the presence of a titanium spinal stabilizer is illustrated in Supplementary-Fig. 1.

Analysis of the bias maps revealed that the DL results showed errors close to zero within both the tumourand the WNL. In contrast, the Input (uncorrected) bias maps demonstrated higher error levels. In SC and ASC tasks, patterns of underdosing the tumourand overdosing the WNL can be seen when using uncorrected dose maps. In AC and ASC tasks, regions close to body surfaces are overdosed in the NC bias maps. The lower error in the DL bias map images demonstrates the strength of DL transformer model in each specific task.

The average ± SD of the voxel-level quantitative metrics for AC, SC and ASC tasks are provided in Table 2.

**Table 2:** The average ± sd of voxel-level quantitative metrics across all tasks

	SSIM (%)	PSNR (dB)	ME (Gy)	MAE (Gy)	RMSE(Gy)	MSE(Gy)	RE (%)	RAE (%)
AC	99.52 ± 0.25	47.28 ± 3.31	-0.026 ± 0.060	0.21 ± 0.09	0.051 ± 0.030	0.004 ± 0.005	3.31 ± 9.90	20.13 ± 6.25
SC	99.88 ± 0.09	55.90 ± 4.00	-0.014 ± 0.050	0.10 ± 0.04	0.040 ± 0.030	0.003 ± 0.005	2.08 ± 9.10	12.32 ± 4.97
ASC	99.57 ± 0.33	47.97 ± 3.60	-0.040 ± 0.060	0.22 ± 0.10	0.055 ± 0.044	0.005 ± 0.007	4.02 ± 12.7	23.8 ± 9.0

**Fig. 4:**
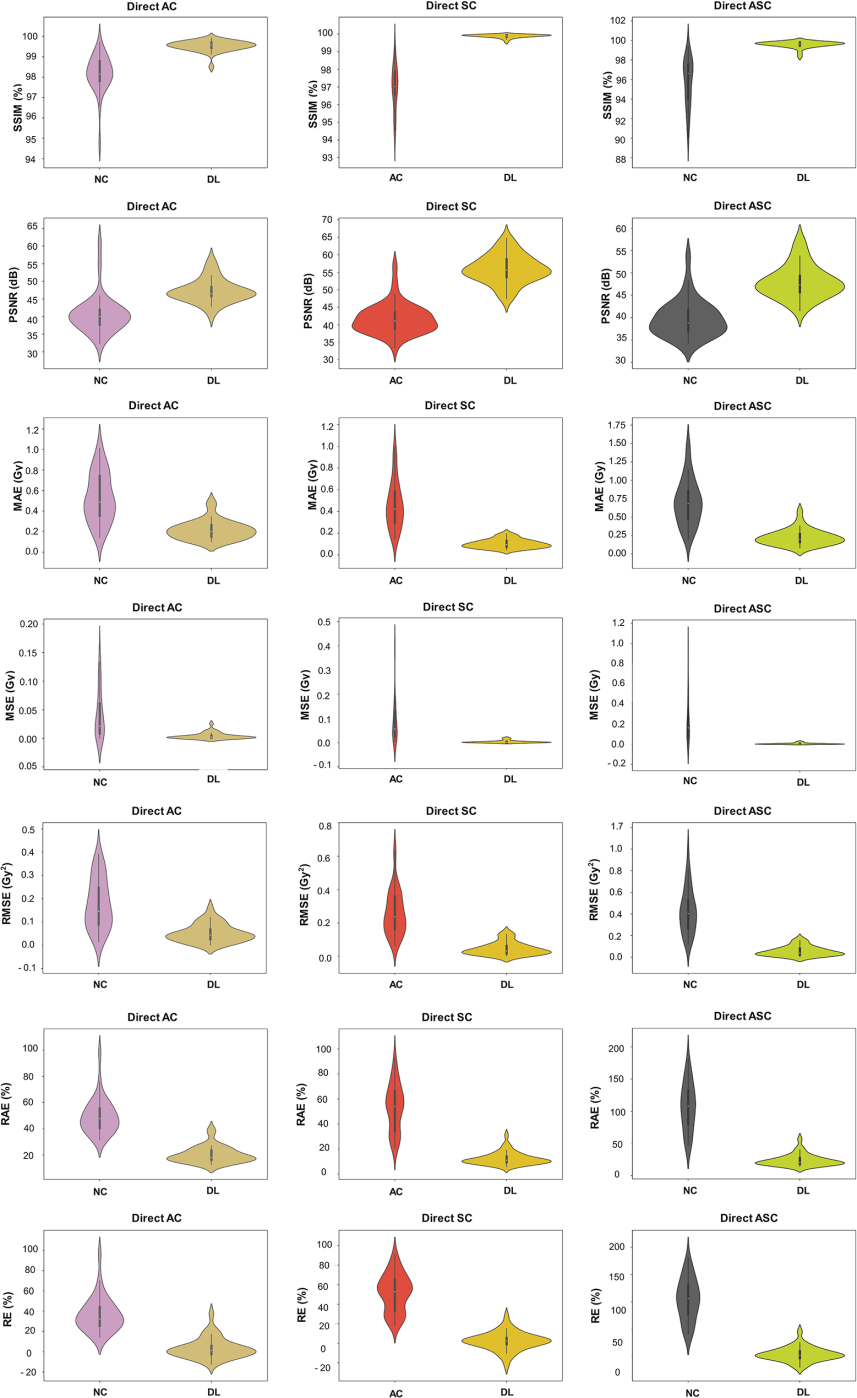
The violin plots show the distribution of quantitative metrics for voxel-level evaluations, displayed from top to bottom as follows: SSIM (%), PSNR (dB), MAE (Gy), MSE (Gy), RMSE (Gy), RAE (%), and RE (%). Metrics from left to right are shown for AC, SC, and ASC tasks

Figure 4 presents the quantitative metrics for voxel-level evaluations calculated for performance evaluation of the models for all tasks.

**Fig. 5:**
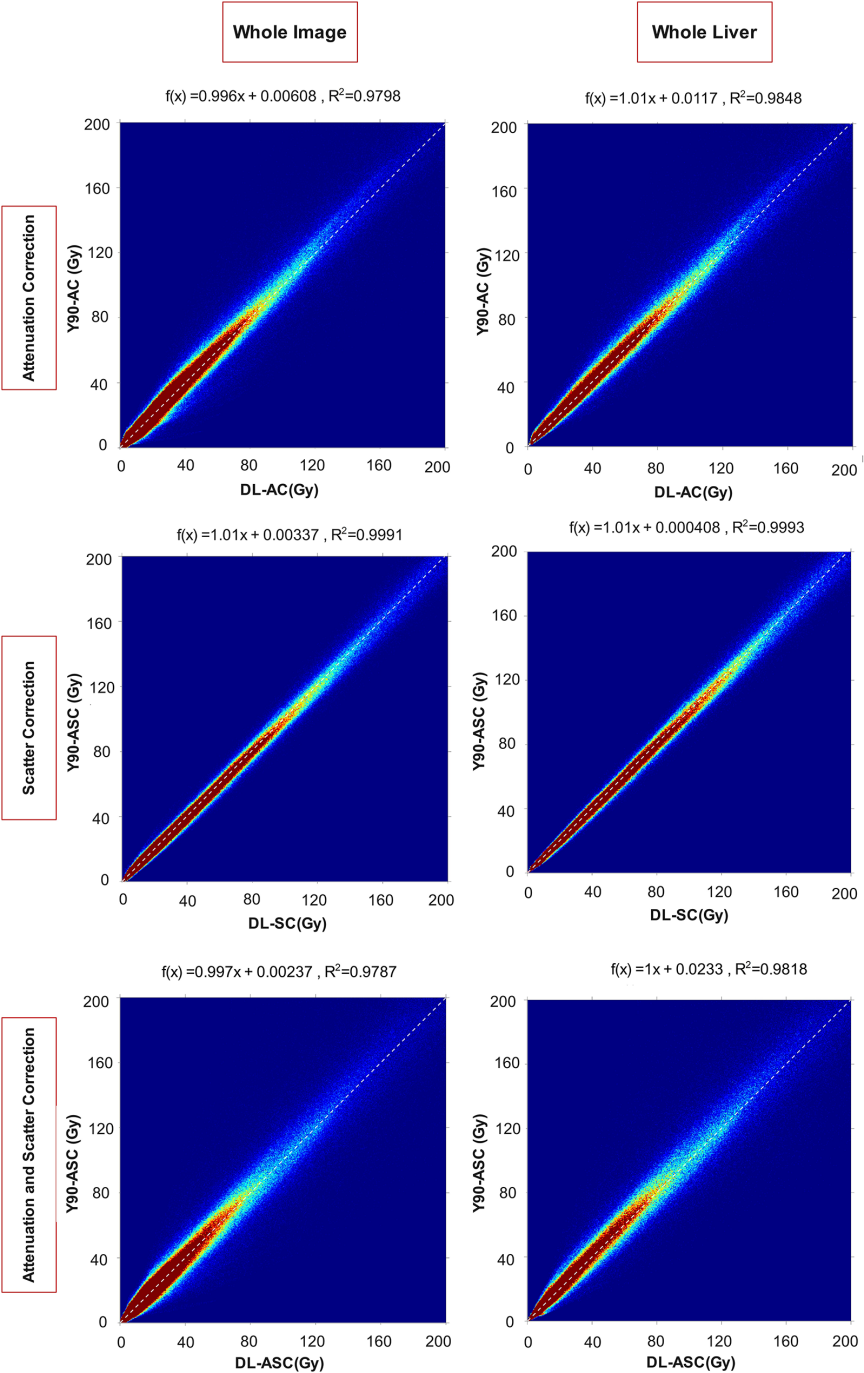
The joint histogram comparing reference dose maps and DL-dose maps for each task is shown, calculated for the whole image (left) and for the whole liver (right)

We also calculated the joint histograms to compare the reference dose maps and DL-dose maps for each task. The joint histograms were once calculated for the whole image and once for the WL. They all show a strong correlation between the reference and DL-dose maps. Figure 5 displays the joint histograms. R^2^ of more than 0.98 for the whole liver voxels shows an excellent match between the DL and reference images. Besides, the slope is very close to 1, showing the minimum over/underestimation by the models.

**Fig. 6:**
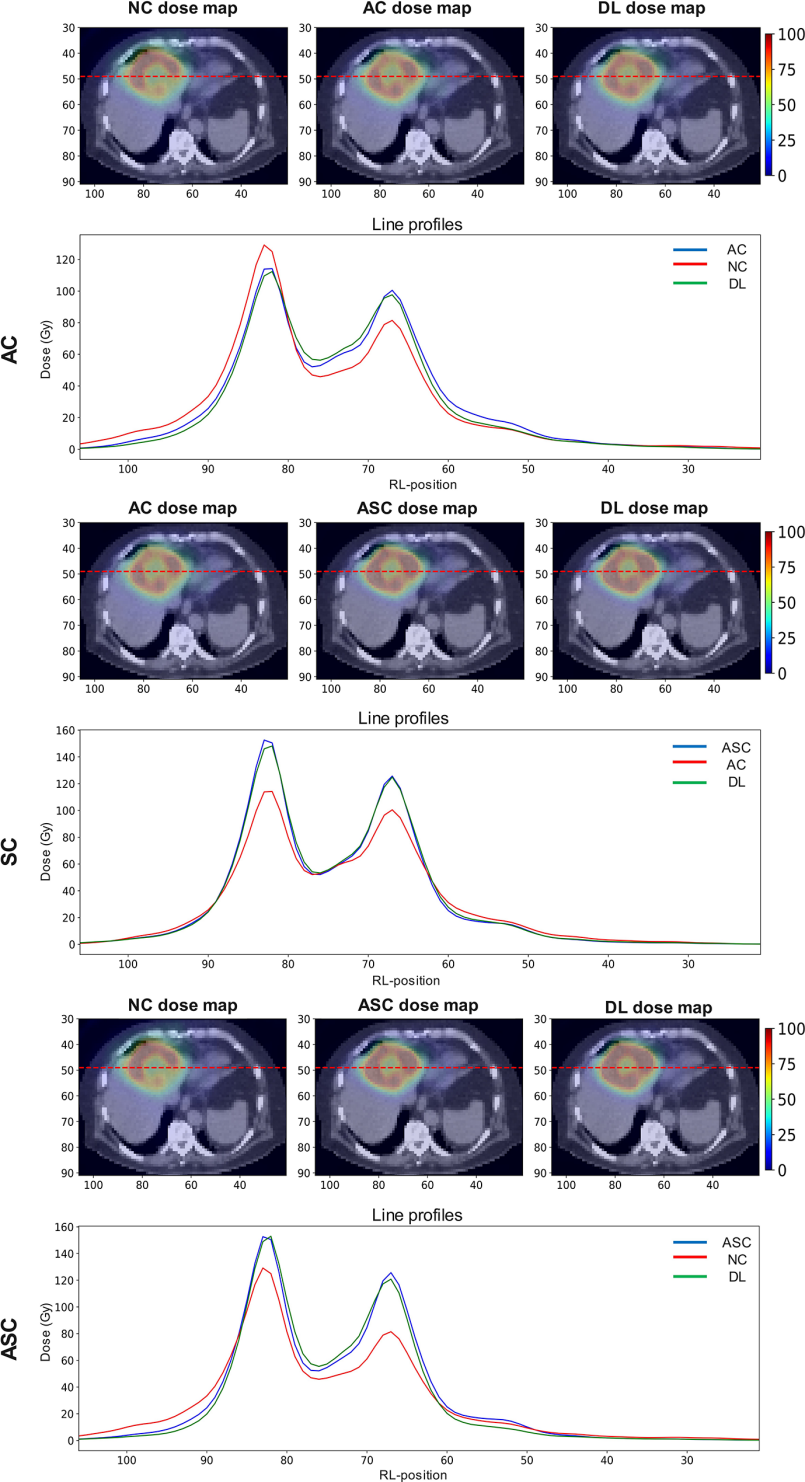
The profiles demonstrate a close alignment between the reference and DL, with observable deviations in the input. The input is represented in red, the reference in blue, and the DL in green. *RL: right-to-left position. All the profiles are drawn on the same axial slice. The top images show the fused CT and dose maps, while the color bar shows the dose map intensity values

Figure 6 depicts the axial (right-to-left) line profiles comparing the input, DL, and reference dose maps. These line profiles are drawn for the same patient at an axial slice in the middle of the tumour. The green line (DL) shows excellent match with the blue line (reference images) both inside and outside the tumoural and liver regions, with the best agreement observed for SC task.

Table 3 summarizes the gamma evaluation pass rates (%) for each task, calculated within the whole image, tumour, and WNL. Additional pass-rate calculations for the lungs, kidneys, and stomach are provided in Supplementary-Table 1. The 4.795 mm and 1% criteria were the most stringent, based on the voxel size of SPECT images. Evaluations were also conducted using other criteria, such as 10 mm and 5%, considering SPECT’s spatial resolution, also 15 mm and 10% due to the lower image quality of ^90^Y bremsstrahlung SPECT images. The highest gamma pass-rates for all three regions, namely the tumour, WNL and whole image, was achieved for SC task.

**Table 3:** Average gamma pass-rates for each task, evaluated using three different DTA (mm) and DD (%) criteria. Gamma pass rates were calculated for the whole body, tumour, and WNL regions

DTA & DD	4.795 mm & 1%				10 mm & 5%				15 mm & 10%			
	Input	AC	SC	ASC	Input	AC	SC	ASC	Input	AC	SC	ASC
Whole image	96.90 ± 2.23	99.04 ± 0.43	99.58 ± 0.26	99.16 ± 0.44	99.45 ± 0.54	99.93 ± 0.05	99.980 ± 0.018	99.92 ± 0.06	99.88 ± 0.13	99.991 ± 0.010	99.998 ± 0.0040	99.988 ± 0.0140
Tumour	96.47 ± 0.86	96.66 ± 0.88	96.79 ± 0.95	96.58 ± 0.87	97.61 ± 0.94	98.80 ± 0.63	98.93 ± 0.99	98.64 ± 0.60	98.77 ± 0.73	99.71 ± 0.30	99.0803 ± 0.4400	99.647 ± 0.282
WNL	96.87 ± 0.87	97.74 ± 0.75	98.30 ± 0.74	97.87 ± 0.87	98.88 ± 0.60	99.56 ± 0.31	99.85 ± 0.15	99.44 ± 0.40	99.688 ± 0.270	99.944 ± 0.074	99.99 ± 0.031	99.907 ± 0.117

### Region-level evaluations

**Fig. 7:**
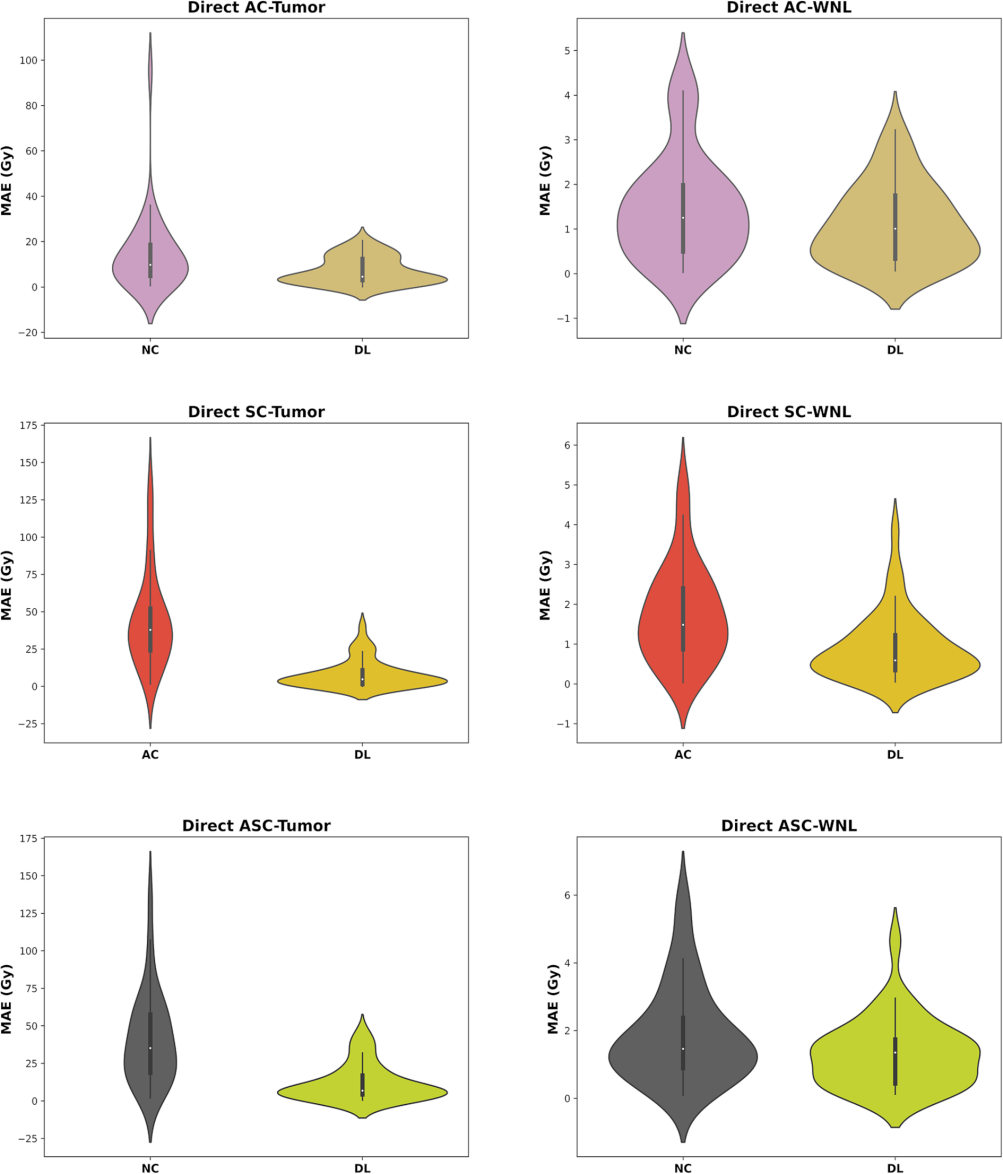
Violin plots of Mean Absolute Error (MAE in Gy) for Input and DL results were generated within the tumour and whole normal liver (WNL) regions. The first row shows MAE for both structures in AC task, the middle row for SC task, and the bottom row for ASC task. The plots indicate that DL-based errors were close to zero, in contrast to the Input images, which showed higher error values

Organ-level evaluations include violin plots that illustrate the distribution of each calculated error. To enhance readability, we only present the MAE (Gy) violin plots for the tumour and WNL in Fig. 7. Other error metrics, such as ME, RAE, RE, and median shift, for the tumor, WNL, kidneys, lungs, and stomach are provided in Supplementary-Figs. 2, 3, 4 and 5. The mean ± SD of the Mean Absolute Error (MAE) in Gy unit for AC task is 7.22 ± 5.90 for the tumor and 1.09 ± 0.86 for WNL. For SC task, these values are 8.0 ± 9.3 and 0.9 ± 0.8, respectively, whereas for ASC task, they are 11.80 ± 12.02 and 1.30 ± 0.98, respectively. These values showed a significant improvement compared to the input images as shown in Fig. 7.

**Fig. 8:**
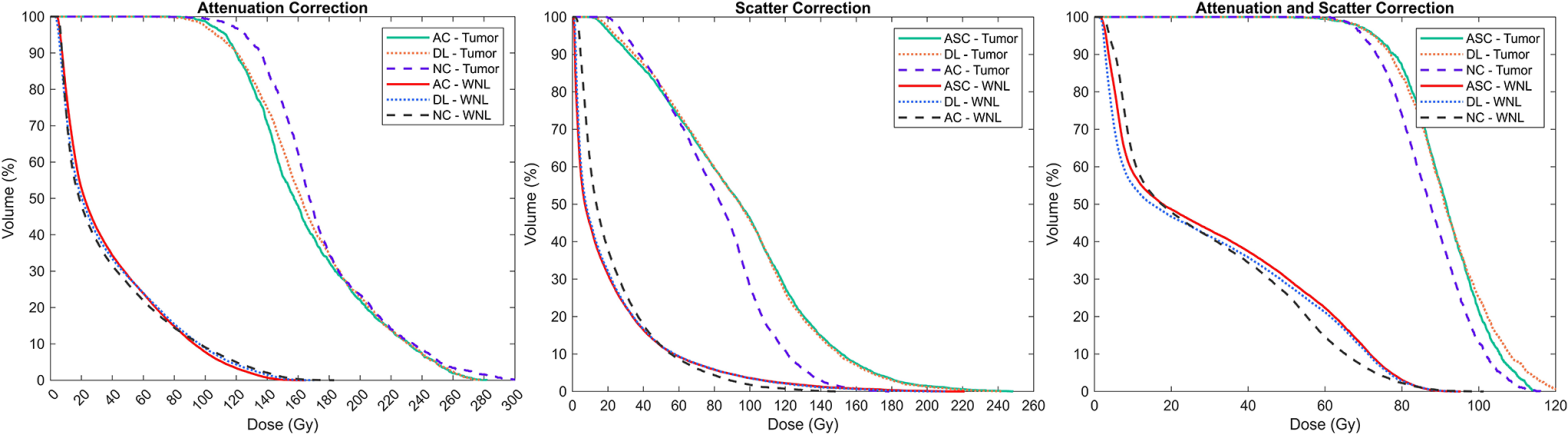
The DVHs for the tumor and WNL were calculated from the Input, Reference, and DL-based dose maps across all tasks. The DL-based DVH (yellow line for the tumor and blue line for the WNL) closely aligns with the Reference DVH (green line for the tumor and red line for the WNL). In contrast, the DVH calculated from uncorrected (Input) images shows noticeable deviations from the Reference, specifically in tumor region

Table 4 summarizes the mean absorbed dose (MAD) ± standard deviation (SD) for each organ based on Reference, Input, and DL-based dose maps, along with the p-values from Mann-Whitney U-test. The DVHs for the tumor and WNL are presented in Fig. 8. We also calculated the DVHs for other organs (kidneys, lungs and stomach) (Supplementary-Fig. 6).

**Table 4:** The mean absorbed dose (MAD) ± standard deviation (SD) for each organ was calculated from the reference, input, and DL-based dose maps. P-values from the Mann-Whitney U-test for each comparison are reported for each organ across all tasks. A p-value > 0.05 indicates no statistically significant difference, whereas a p-value < 0.05 indicates a statistically significant difference

Task	Organs	Reference (MAD ± SD)	Input ^90^Y (MAD ± SD)	*P*-value (input- ^90^Y)	DL (MAD ± SD)	*P*-value (DL)
AC	Tumour	154.91 ± 63.94	154.27 ± 74.4	> 0.05	148.58 ± 62.06	> 0.05
	WNL	39.28 ± 19.75	38.27 ± 19.24	> 0.05	38.34 ± 19.25	> 0.05
	Lungs	5.32 ± 3.05	9.07 ± 5.37	**< 0.05**	6.47 ± 4.11	> 0.05
	Kidneys	10.63 ± 7.98	9.20 ± 6.91	> 0.05	10.37 ± 7.77	> 0.05
	Stomach	9.36 ± 6.30	8.46 ± 4.90	> 0.05	9.47 ± 6.15	> 0.05
SC	Tumour	209.41 ± 102.23	165.72 ± 73.44	**< 0.05**	205.35 ± 94.39	> 0.05
	WNL	40.01 ± 20.60	41.28 ± 19.93	> 0.05	40.04 ± 20.86	> 0.05
	Lungs	5.07 ± 2.84	5.63 ± 2.95	> 0.05	4.80 ± 2.78	> 0.05
	Kidneys	6.85 ± 5.80	10.57 ± 7.46	**< 0.05**	6.87 ± 5.90	> 0.05
	Stomach	6.19 ± 5.27	8.95 ± 5.93	**< 0.05**	6.24 ± 5.80	> 0.05
ASC	Tumour	195.13 ± 90.55	154.27 ± 73.38	**< 0.05**	186.32 ± 83.53	> 0.05
	WNL	37.90 ± 20.61	38.27 ± 19.24	> 0.05	37.10 ± 20.41	> 0.05
	Lungs	4.83 ± 3.03	9.08 ± 5.37	**< 0.05**	5.50 ± 4.07	> 0.05
	Kidneys	6.99 ± 6.31	9.21 ± 6.91	> 0.05	6.64 ± 6.05	> 0.05
	Stomach	6.55 ± 5.63	7.03 ± 3.63	**< 0.05**	5.06 ± 3.88	> 0.05

The results of testing the AC model on the calibration phantom and external patient acquired on the GE NM 860 camera are provided in Supplementary-Fig. 7. The voxel-level RE (%) and RAE (%) for the phantom were 14.54% and 18.5%, respectively. We also segmented the internal space of the phantom and both the region-wise RE (%) and RAE (%) were 13.03%. The RE (%) and RAE (%) within the liver of external patient were − 6.48%, and 6.48%, region-wise, and − 7.24%, and 10.23% voxelwise, respectively. Also, plots of the loss and accuracy curves for each fold in all three tasks are provided in Supplementary-Fig. 8.

## Discussion

This study addresses the challenges of quantitative ^90^Y bremsstrahlung SPECT/CT imaging by implementing CT-free attenuation and Monte Carlo-based scatter corrections. These techniques were used to generate absorbed dose maps from ^90^Y-SPECT images, providing a reliable ground truth for training deep learning models to enhance and accelerate quantitative imaging-guided dosimetry in SIRT. A notable strength of this study is the relatively large cohort size along with the extensive evaluations of model performance at both organ and voxel levels, which is the ultimate goal of ^90^Y SPECT/CT imaging in the era of personalised treatment.

To the best of our knowledge, this is the first implementation of such corrections in the dose domain. The rationale for using dose maps as both the input and output of the models is rooted in the meaningful and consistent nature of dose intensity values (Gy) in these maps. Notably, we used bSPECT images directly to train models across various tasks. The results are provided in Supplementary-Tables 2 and 3. A primary reason for suboptimal results lies in the fact that SPECT images are count-based, and the quantities lack inherent physical significance. This fact may even limit the generalizability of models trained in the image domain as the normalisation values and voxel intensity range on an external image depends on the sensitivity of the scanner, acquisition time, number of azimuths and the injected activity. However, the voxel values have an interpretable and meaningful range when we convert the images to dose maps. The inferior results of the training in the bSPECT image domain compared to the main results of the dose domain training reported in the manuscript supports our hypothesis. Additionally, the inherently noisy nature of ^90^Y bSPECT images further complicates the modelling process.

Our organ-level includes not only tumors and WNL, but also other organs, such as the lungs, stomach and kidneys to assess the model’s correction performance across the entire image. This allows us to evaluate the model robustness and consistency of the quality of corrections in various tissue types. Definitely, the delivered dose to these organs is also important due to safety concerns. This study therefore represents a significant contribution toward improving the accuracy and efficiency of ^90^Y SIRT dosimetry, with potential implications for clinical adoption and optimization in personalised radionuclide therapy.

The most accurate approach for modelling attenuation and scatter is MC simulation. However, the computational burden of MC simulations hampers routine use in the clinic. Notably, even commonly used non-MC methods can considerably increase reconstruction time. One possibility is using GPUs for MC simulations, which is challenging as mentioned in the “Introduction” section and further elaborated in [23]. Our primary objective in training these models was to address the computational burden of MC simulations. Furthermore, we aimed to develop models that could support centers lacking GPU-based corrections or centers equipped with standalone SPECT cameras, thus enabling them to improve or establish ^90^Y-SIRT procedures more effectively. To this end, we used high-quality reference dataset for training, which were corrected for attenuation using low-dose CT scans, and for scatter using Monte Carlo-based scatter correction. Additionally, to avoid inaccuracies in CT-based attenuation correction due to CT artefacts, we refined the dataset by excluding cases affected by these artefacts. This approach allowed us to enhance the accuracy of attenuation correction by training the model on clean, artefact-free and Monte Carlo SC data.

We utilized a GPU-based scatter correction tool, “the HybridRecon system”, for MC-based scatter correction, which is already highly efficient. However, this process can take over an hour on a CPU, with reports indicating a runtime of approximately 80 min [32]. In contrast, our deep learning models achieved inference times of about 20 s per SPECT image on a dedicated GPU and approximately 6 min per SPECT image using an Intel Core i9-13900KF CPU.

We compared the DL results and Reference images in both voxel and organ level dosimetry analysis. To this end, we opted to perform voxel-level dosimetry using the local energy deposition method for several reasons. First, this method provides sufficient accuracy for dosimetry of a beta emitter, such as ^90^Y. Second, the local energy deposition method incorporates patient-specific calibration for quantification, using a conversion factor to translate count measurements into activity and consequently to absorbed dose values. This approach produces dose maps that closely resemble the original image data, which are different only by the applied conversion factor. Therefore, this approach not only preserves the fundamental characteristics of the original images but also improves the interpretability of their values, similar to standardized uptake value (SUV) conversions in PET imaging, albeit not directly comparable.

We adopted Swin UNETR, a state-of-the-art DL architecture that combines the UNet-inspired encoder-decoder structure with transformer layers and attention mechanism in encoder, and deep supervision leveraging the strengths of both. This architecture learns through a multi-scale, patch-wise attention mechanism, using patches or windows in multiple sizes and shifting them across the entire image. This feature allows the model to capture fine details while also keeping an eye on the global patterns. Such capability might be beneficial for this study given the noisy and degraded nature of ^90^Y-SPECT images. The network can ignore noise and artefacts and retain relevant signals only, which may enhance its performance in correction tasks. Additionally, the window-shifting nature of this architecture enables to achieve good outcomes without overburdening computational resources. In addition, we used patch-based training and sliding windows inference for testing our models during inference. This approach tends to make the models more robust against changes in voxel dimensions, field-of-view and patient size compared to approaches using image resizing. We used five different models on each external data and averaged the output, in this way each image compensates for the biases in each model to reproduce the pattern in the training data.

In the present study, the models were trained for three tasks: AC, SC, and ASC, keeping attenuation and scatter components separate and combined, each yielding promising results. Notably, as shown in Fig. 3, SC and ASC tasks using either AC or uncorrected (NC) images tend to overestimate dose values in normal liver regions and underestimate them in the tumors. This trend is also observed in line profiles (Fig. 6), where high-dose regions (tumors) are underestimated without SC. These results are aligned with results from other studies [20]. Additionally, organ-wise assessments in Table 3 reveal a statistically significant difference in “tumor absorbed dose” between the input and reference dose maps for both ASC and SC tasks. In contrast, in ASC and AC tasks, a significant statistical difference is observed in “lung absorbed dose” calculations. Although lung-shunt calculations are performed as pre-therapy procedure with ^99m^Tc-MAA planning study, the lung dose calculations post-therapy is of importance for potential toxicity evaluation.

The results highlight how our DL models are sensitive to the presence of high-density objects and heterogeneous tissue compositions, which directly affect attenuation and scatter correction in SPECT imaging. Metal implants, such as titanium spinal stabilizer in Supplementary-Fig. 1, introduce severe photon attenuation and beam-hardening effects, leading to localized signal dropouts and distortion of the reconstructed activity distribution. Since our DL models were not trained with sufficient examples of such high-density foreign objects, they struggled to correct these artifacts, resulting in decreased performance. Similarly, calcifications in the hepatic vasculature and air-tissue interfaces (e.g., tumors adjacent to gastrointestinal gas or lung regions) introduce complex scattering patterns. AC and SC processes experience misestimations due to interfaces’ sudden changes in attenuation coefficients. Attenuation is reduced when air is present in the field-of-view. In contrast, excessive localized attenuation can be produced by high-density calcifications, both of which impair model generalizability.

We extensively tested the models through various dosimetric analyses on a test dataset that remained unseen by the models. We performed region- and voxel-wise analyses, DVH evaluations, and Gamma pass rate, with different criteria and other metrics to thoroughly assess model performance. The aim was to identify the strengths and limitations of each model, providing end users with insight into model robustness. Three distinct models were developed for use in different clinical scenarios. However, as the dataset was sourced from a single center and scanner, model generalizability remains uncertain. This limitation might introduce bias inherent to the specific institution, such as imaging protocols, scanner types, or patient demographics. These factors could potentially affect the model’s ability to generalize effectively when applied to data from other centers or different clinical settings. We aim to address this concern in future studies by including data from multiple centers with different scanners and patient populations. To address this, future training with multicenter cohorts is planned. Once this is completed, clinical use of the models will be feasible. Additionally, separating and combining attenuation and scatter correction in this study facilitates model integration with commercial reconstruction software for ^90^Y-SPECT. The trained models and inference instructions will be publicly available on the lab’s GitHub page after publication.

## Conclusion

Swin-UNETR models were trained for CT-free attenuation correction and/or MC-based scatter correction, as separate or combined tasks, in a fraction of the time, for ^90^Y dose maps calculated from SPECT/CT images or SPECT images only. A relatively large cohort was reconstructed for attenuation and/or scatter correction using CT-based and Monte Carlo-based scatter correction using the Hermes reconstruction tool (Hybrid Recon™), serving as standard of reference for our DL model. Evaluation of the performance of these models at the organ- and voxel-levels showed comparable performance to a commercial reconstruction tool. Such models are promising for clinical applications, particularly in centers equipped with standalone SPECT cameras or those without access to GPUs for MC-based corrections after training with a larger dataset.

## Electronic supplementary material

Below is the link to the electronic supplementary material.

Supplementary Material 1

## Data Availability

The data used in this work is not available.

## Abbreviations

SIRT: Selective internal radiation therapy
bSPECT: Bremsstrahlung SPECT
PET: Positron emission tomography
CNN: Convolutional Neural Network
WNL: Whole normal liver
DEW: Dual energy window
TEW: Triple energy window
MC: Monte Carlo
GPU: Graphics processing units
MAA: Macroaggregated albumin
NC: No attenuation and scatter correction
AC: Attenuation correction
ASC: Attenuation and scatter correction
OSEM: Ordered Subsets Expectation Maximization
SC: Scatter correction
SSIM: Structural similarity index
RMSE: Root mean squared error
PSNR: Peak signal-to-noise ratio
MAE: Mean absolute error
ME: Mean error
RE: Relative error
RAE: Relative absolute error
DTA: Distance-to-agreement
DD: Dose-difference
OAR: Organ-at-risk
WL: Whole liver
MAD: Mean absorbed dose
DVH: Dose-volume histograms
SD: Standard deviation
DL: Deep learning
RAM: Random Access Memory

## Code availability

The data used in this work are not available. These models will be made available on Github.
