# Supplementary material for: CT-free attenuation and Monte-Carlo based scatter correction-guided quantitative 90Y-SPECT imaging for improved dose calculation using deep learning
Source: Eur J Nucl Med Mol Imaging. 2025 Mar 13;52(9):3484–99. doi: 10.1007/s00259-025-07191-5 (PMC12222404; doi:10.1007/s00259-025-07191-5)
Supplement: Supplementary file 1 — Supplementary Material 1 [file 259_2025_7191_MOESM1_ESM.pdf]

Supplemental section

Supplemental-Table 1. Average gamma pass-rates for each task, evaluated using three different DTA (mm) and DD (%) criteria. Gamma pass rates were calculated for the whole lungs, Stomach, and Kidney regions.

DTA & DD	4.795 mm & 1%				10mm & 5%				15mm & 10%			
	Input	AC	SC	ASC	Input	AC	SC	ASC	Input	AC	SC	ASC
**lungs**	97.81 ± 1.27	98.65 ± 0.8	99.46 ± 0.5	99.03 ± 0.75	99.46 ± 0.64	99.86 ± 0.24	99.99 ± 0.03	99.91 ± 0.172	99.884 ± 0.227	99.98 ± 0.08	100 ± 0.001	99.984 ± 0.047
**Stomach**	97.36 ± 1.24	98.72 ± 0.85	99.56 ± 0.68	98.88 ± 1.24	99.76 ± 0.413	99.95 ± 0.11	99.98 ± 0.12	99.95 ± 0.16	99.99 ± 0.049	100 ± 0.002	99.998 ± 0.01	99.996 ± 0.019
**Kidneys**	97.76 ± 1.18	99.014 ± 0.87	99.53 ± 0.66	99.12 ± 0.95	99.46 ± 0.64	99.93 ± 0.14	99.99 ± 0.015	99.94 ± 0.11	99.9 ± 0.245	99.994 ± 0.025	100	99.996 ± 0.014

Supplementary-Table 2. Voxel-level quantitative metrics when using bSPECT images for training the models across all tasks.

	SSIM (%)	PSNR (dB)	ME (Gy)	MAE (Gy)	RMSE(Gy)	MSE(Gy)	RE (%)	RAE (%)
**AC**	99.5± 0.3	47± 3.6	-0.84± 0.4	0.85 ± 0.4	0.84 ± 0.4	0.87 ± 0.83	-44.4± 7.4	45.8 ± 6.89
**SC**	99.88± 0.11	56.06 ± 4.87	-0.61± 0.3	0.61 ± 0.3	0.61 ± 0.3	0.46 ± 0.4	-35.17 ± 9.1	35.96 ± 8.5
**ASC**	99.6 ± 0.25	47.88 ± 4.02	-0.6± 0.3	0.6 ± 0.3	0.6 ± 0.3	0.45 ± 0.5	-34.41 ± 8.7	38.5 ± 7.05

Supplementary- Table 3. Organ -level quantitative metrics when using bSPECT images for training the models across all tasks.

organ	task	RE%	RAE%	ME(Gy)	MAE(Gy)	Median-Shift (Gy)
**Tumor**	**AC**	-46.42 ± 7.82	46.42 ± 7.82	-72.6 ± 34.01	72.6 ± 34.01	-66.26 ± 29.3
	**SC**	-37.66 ± 7.4	37.66 ± 7.4	-78.98 ± 40.38	78.98 ± 40.38	-71.92 ± 35.66
	**ASC**	-37.12 ± 8.3	37.12 ± 8.3	-73.19 ± 39.7	73.19 ± 39.7	-63.89 ± 31.63
**WNL**	**AC**	-45.91 ± 6.64	45.91 ± 6.64	-17.8 ± 9.23	17.8 ± 9.23	-14.12 ± 9.23
	**SC**	-36.77 ± 7.45	36.77± 7.45	-14.59± 7.98	14.59 ± 7.98	-9.44 ± 7.43
	**ASC**	-36.58 ± 6.95	36.58 ± 6.95	-13.76 ± 7.97	13.76 ± 7.97	-9.59 ± 7.76

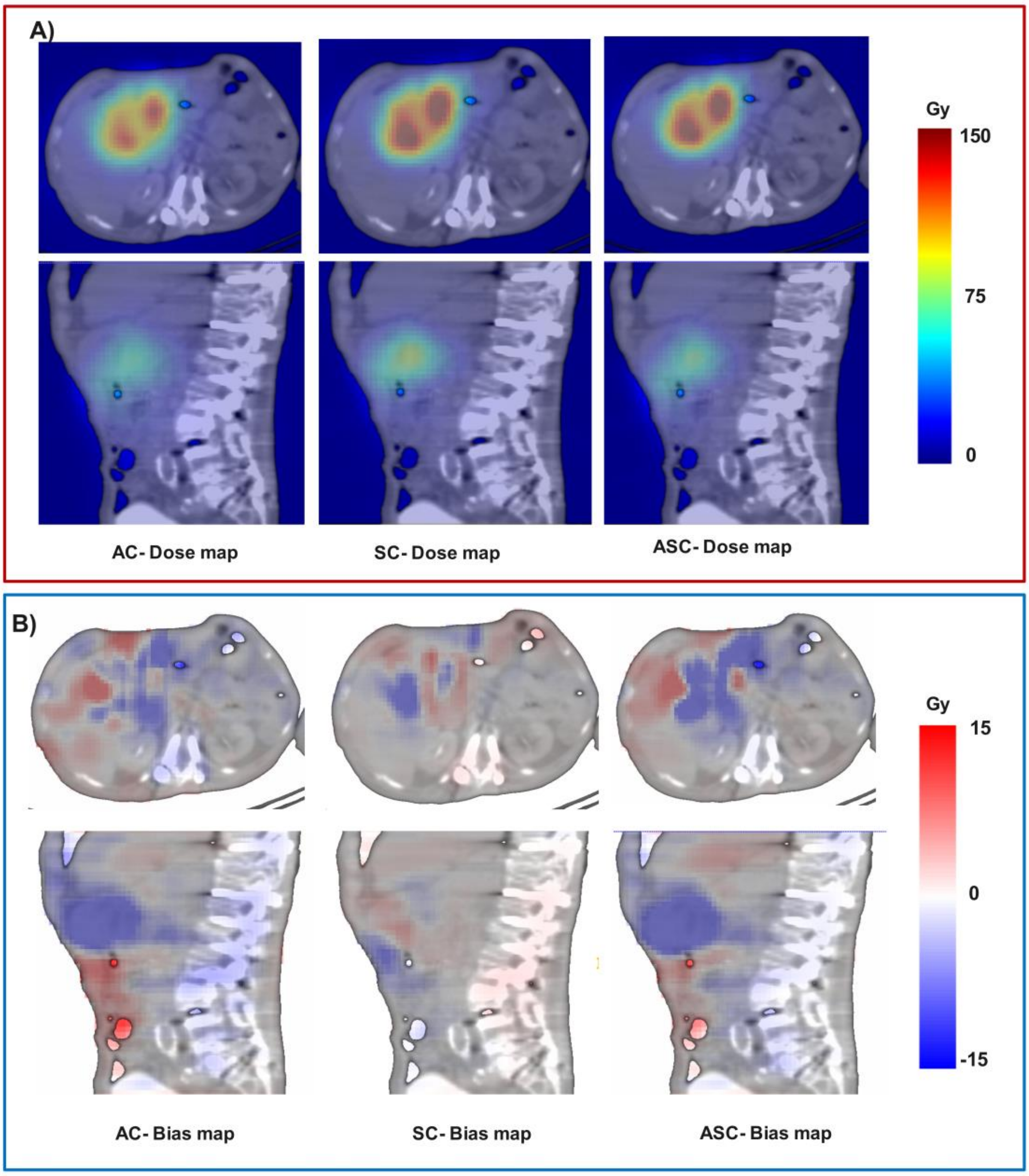

Supplementary-Figure 1. Case illustrating an instance where our DL model has limited performance. Panel A presents the DL model's results for each task, while Panel B displays the corresponding bias maps relative to the reference. The patient in this case had a titanium spinal stabilizer implant, a metal object unfamiliar to our DL models, which contributed to decreased performance. The relative absolute errors for this case are 26.8%, 22.81%, and 34.66% for AC, SC, and ASC tasks, respectively

.

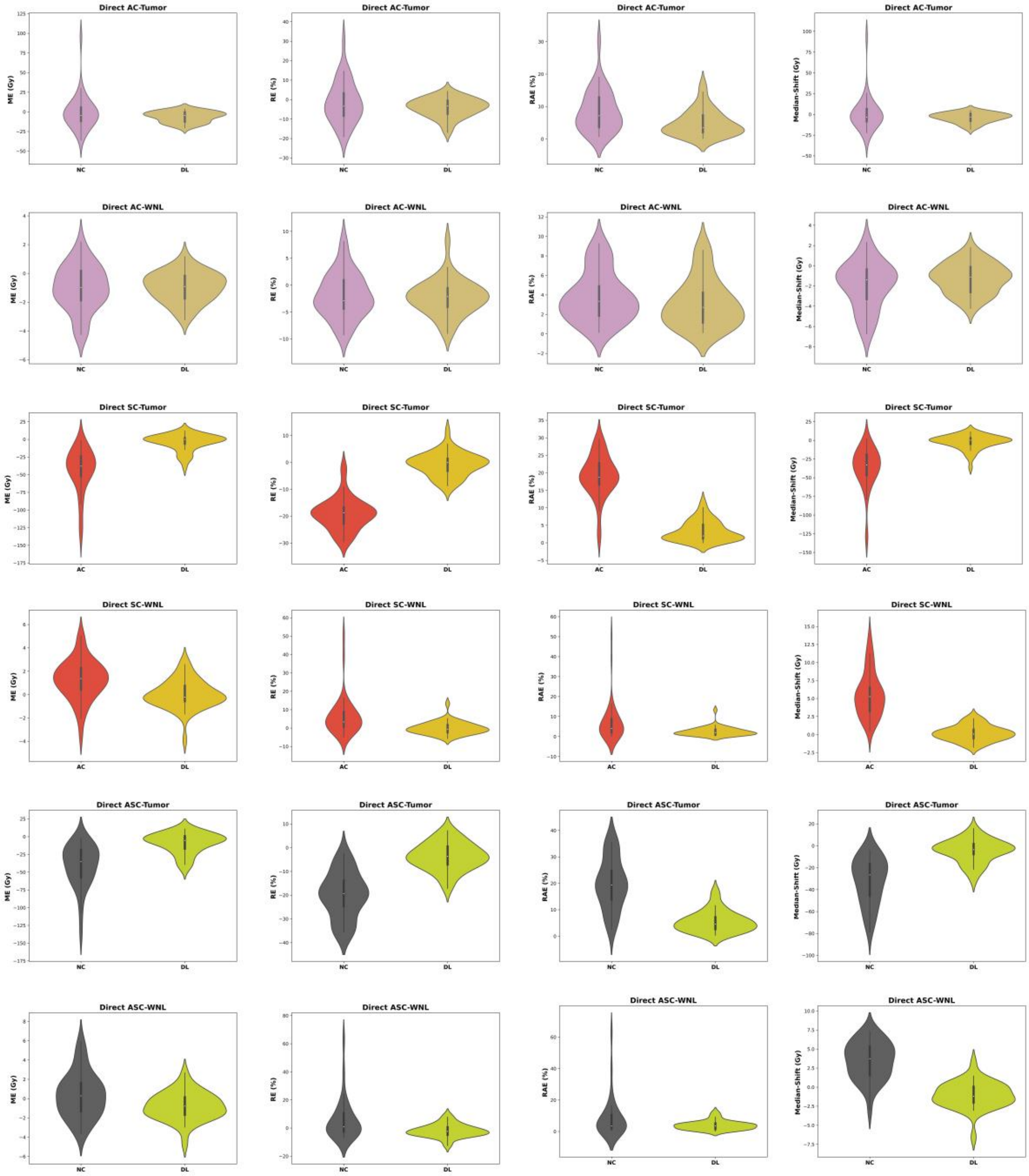

Supplementary-Figure 1. Violin plots of other errors for Input and DL-based images were generated within the tumor and whole normal liver (WNL) regions across all tasks.

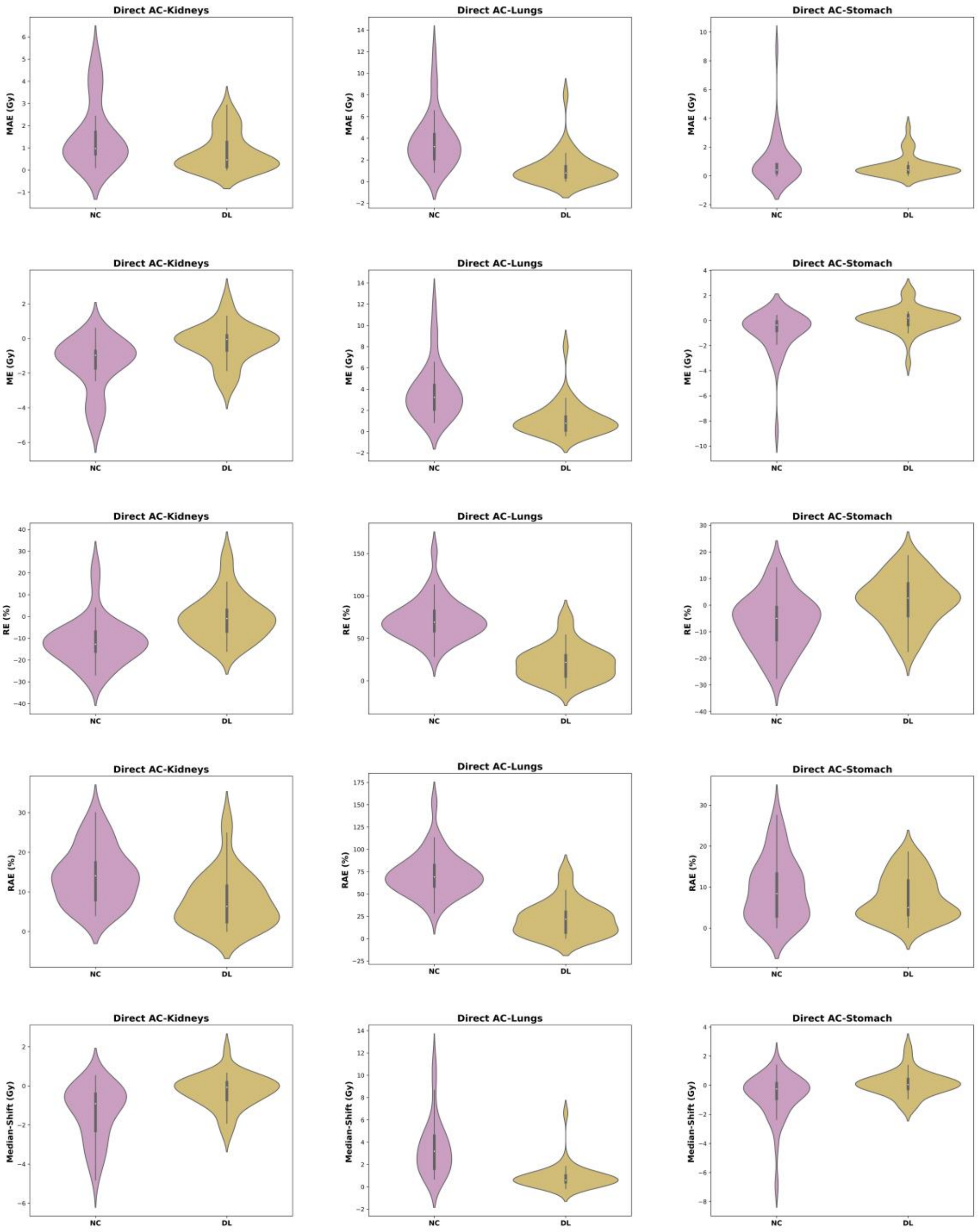

Supplementary -Figure 2. Violin plots of Errors for Input and DL-AC-based images were generated within the Kidneys, lungs, and stomach regions.

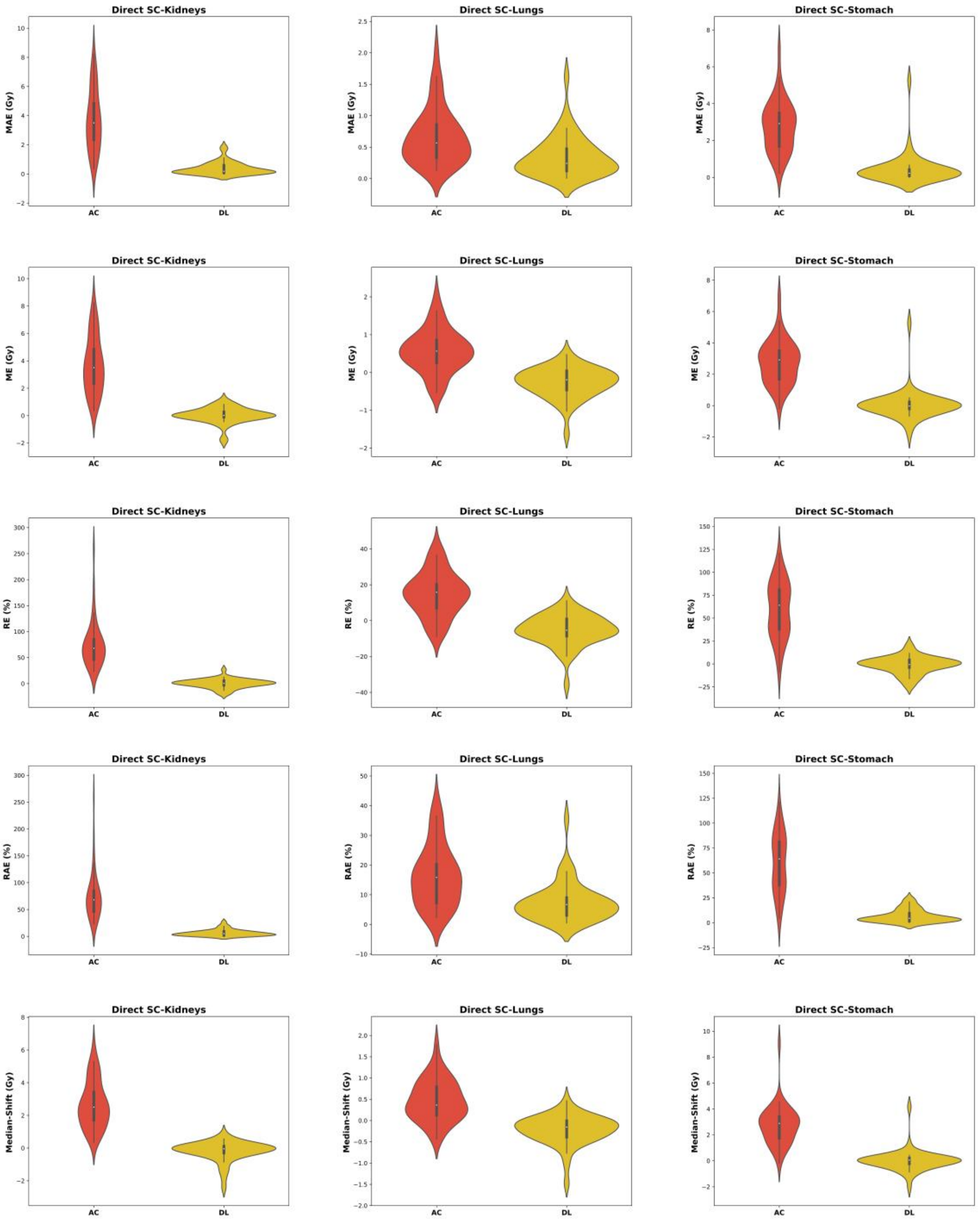

Supplementary -Figure 3. Violin plots of Errors for Input and DL-SC-based images were generated within the Kidneys, lungs, and stomach regions.

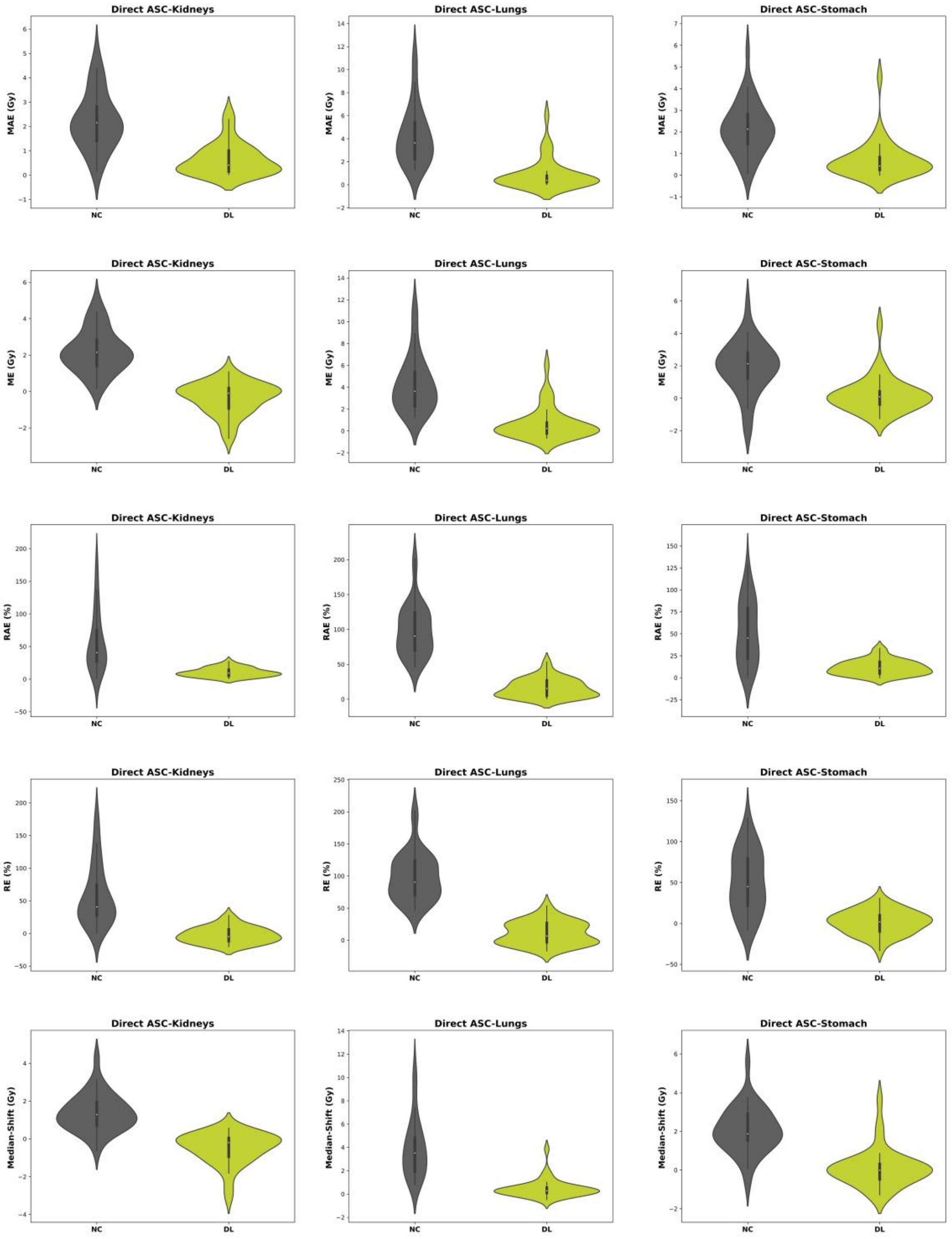

Supplementary -Figure 4. Violin plots of Errors for Input and DL-ASC-based images were generated within the Kidneys, lungs, and stomach regions.

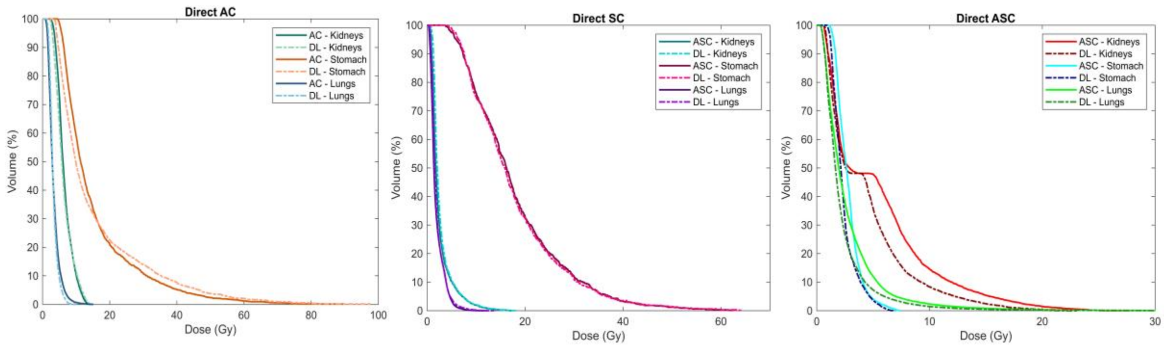

Supplementary -Figure 5. The DVHs for the kidneys, lungs, and stomach were calculated from the Input, Reference, and DL-based dose maps across all tasks.

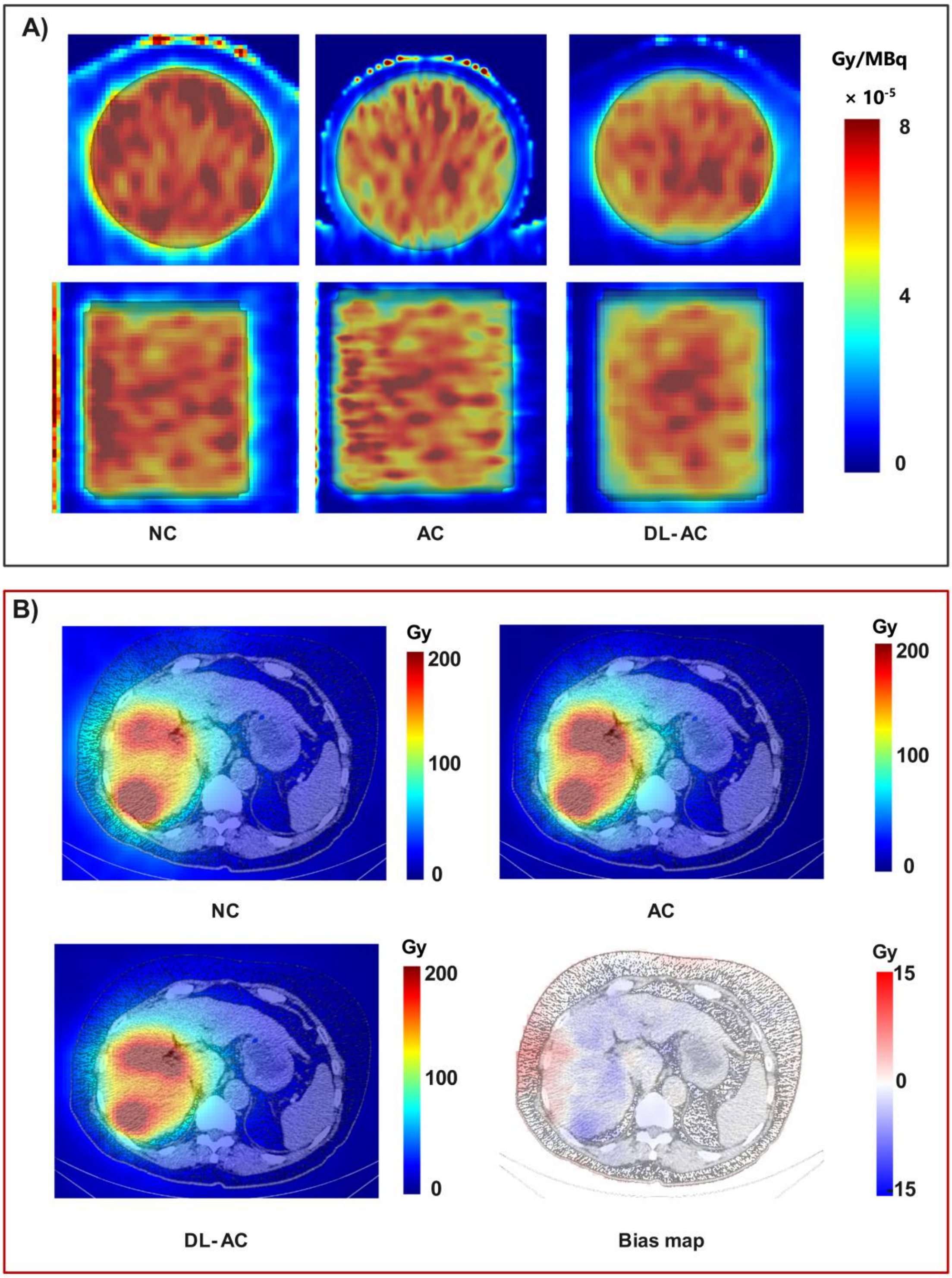

Supplementary-Figure 6. Inference of the AC model on A) the calibration phantom, achieving voxel-level RE (%) and RAE (%) of 14.54% and 18.5%, respectively, and 13.03 % region-wise. Panel B) External dataset from GE NM 860 SPECT/CT camera with different spacing than our training dataset. The RE (%) and RAE (%) within the liver were -6.48%, and 6.48%, region-wise, and -7.24%, and 10.23% voxel wise, respectively.

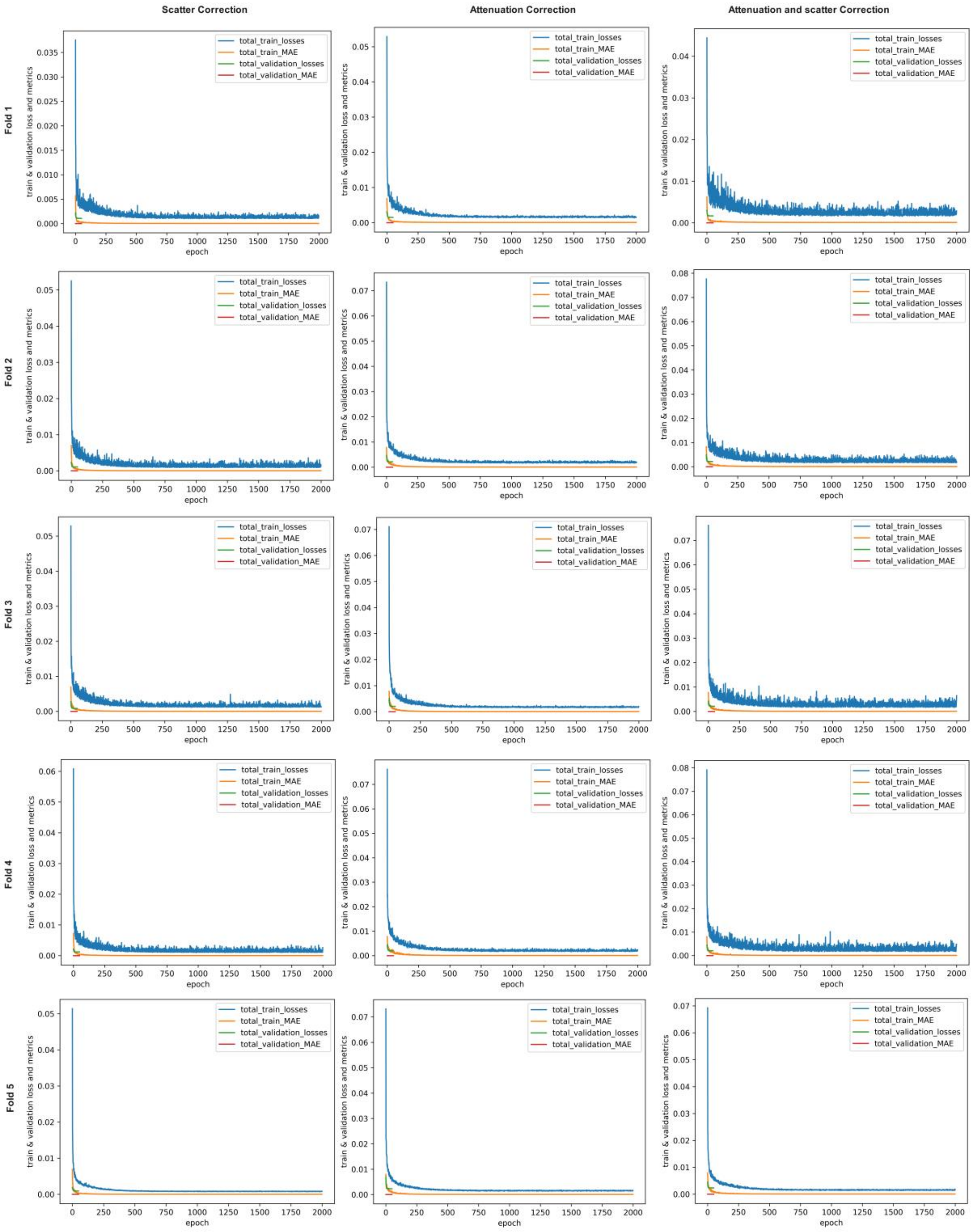

Supplementary- Figure 7. Plots of the loss and accuracy curves.
